# Dr. McCormack to a Baltimore Audience

**Published:** 1907-09

**Authors:** 


### DR. McCORMACK TO A BALTIMORE AUDIENCE.

One-third of the people sick in this State in 1905, and every year, and one-third of those you took to your cemeteries were sick and died of diseases which your medical profession could and would have prevented if it could have had the intelligent co-operation of your people. You had in that year 2388 deaths from consumption, which means that you have about 8000 cases of this disease in your State constantly. The common impression is that this is an inherited malady, but this is an error. No matter what your mother or father died of, you can no more have consumption except by getting in your body the germs of a previous case than you can raise corn or wheat on one of your rich Maryland farms without seed. If all the expectorated matter and other infectuous discharges from every case now in your State could be collected and destroyed until the victims recovered or died, there need never be another case within your borders unless it be an imported one. You had 1442 deaths from the diseases of children caused by using dirty, adulterated or spoiled milk.

We often speak of the slaughter of the innocents by Herod, but he was a novice in the business as compared with our modern cities. You shuddered with horror over the loss of life on the Larchmont and in the New York Central wreck the other day, and properly so, but more babies die needlessly every week in your own State during the hot season that there were people killed in both of these disasters, and it goes on almost without comment. And it would be cheaper for you to inspect the dairies or sterilize the milk, and save these babies than it would to bury them. You had 256 deaths from diphtheria and scarlet fever, all distinctly preventable. You had 476 deaths from typhoid fever during that year. This means, according to the best estimates, that you have about 6000 cases of this disease during each year.

Now, a State has no more valuable asset than that represented in its healthy population. According to the political economists, to say nothing of the cost of caring for the sick who recovered from these diseases, this represents a distinct loss to your people of $5,848,000. —Maryland Medical Journal, June, '07.

